# Modeling Cost-Effectiveness of Universal Varicella Vaccination With Different Varicella Vaccines in the United Kingdom

**DOI:** 10.1093/cid/ciab040

**Published:** 2021-01-28

**Authors:** Manjiri Pawaskar, Colleen Burgess, Matthew Pillsbury, M Nabi Kanibir, Heather L Platt

**Affiliations:** 1 Merck & Co, Kenilworth, New Jersey, USA; 2 MSD, International, Luzern, Switzerland

To the Editor—We read with interest the article by Akpo et al [1] comparing the cost-effectiveness of varicella vaccination in the United Kingdom (Varilrix, Priorix-Tetra, GSK, Belgium [V-GSK] and Varivax, ProQuad, Merck & Co, Inc, Kenilworth, NJ, USA [V-MSD]). This is an important contribution to the literature demonstrating value of varicella vaccination; however the use of predicted efficacy inputs for 1-dose V-MSD may not accurately reflect the actual vaccine performance and cost-effectiveness, considering availability of observed efficacy and effectiveness data.

Efficacy inputs are among the key drivers of the cost-effectiveness of any intervention. The authors derive 1-dose efficacy inputs of 78% for V-MSD from a methodological study using a statistical model [2] relating immunogenicity data (varicella-zoster virus antibody titer, >5 glycoprotein enzyme-linked immunosorbent assay units per milliliter, 6 weeks after vaccination) to long-term disease breakthrough. The efficacy estimate reported [2] was based on antibody titer with predicted efficacy of 94.0% for all ages and 87.2% in younger children (n = 326; median age, 13 months). However, Akpo et al [1] used predicted efficacy of 78% from sensitivity analysis that was included to illustrate the impact of a 2-fold decrease in antibody titer on efficacy (from 88% to 78%) in children who were vaccinated at age 18 months.

While immunogenicity data can be used as a correlate of protection, using predicted efficacy based on antibody titers alone is a limitation given actual efficacy data is available for V-MSD. Several randomized control trials (RCTs) and observational studies have been published, demonstrating the long-term efficacy [3–6] and effectiveness of V-MSD [7–9]. Kuter et al [3], in an RCT with 10 years of follow-up, showed that 1-dose efficacy of V-MSD was 94.4% (95% confidence interval, 92.9%–95.7%), and 2-dose efficacy was 98.3% (97.3%–99.0%).

Akpo et al [1] did not include the data showing higher efficacy of 1-dose V-MSD [3], with the rationale that this RCT was conducted in children aged 12 months to 12 years and noting that older children may experience a lower risk of infection. However, the average age in this RCT was 4.43 years, supporting efficacy in younger children. Another RCT showed that the seroconversion rates for V-MSD by age groups were comparable—98% for age 12–15 months, 97% for 16–23 months and 2–4 years, and 95% for 5–12 years—with efficacy of 86% for all ages [4]. Two other RCTs (average age of children, 3.6 years and 15 months) showed 1-dose efficacy of V-MSD of was 88.5% and 90.5%, respectively [5, 6]. Similarly, literature reviews, meta-analyses and surveillance studies with up to 14 years of follow-up have shown 1-dose effectiveness for V-MSD ranging from 81% to 100%, depending on disease severity [7–9] (Table 1).

**Table 1.** Summary of Key Literature on the Efficacy and Effectiveness of Varivax Vaccine (1 or 2 Dose)

Study	Varivax Vaccine, 1 or 2 Dose	Study Design	Patient Ages, Range (Mean)	Follow-up Period, y	Efficacy/Effectiveness, %
Kuter et al (2004) [3]	Both 1 and 2 dose	RCT	12 mo to 12 y (4.4 y)	10	1 Dose: 94.4 (95% CI: 92.9– 95.7); 2 doses: 98.3 (97.3–99.0)
White et al (1991) [4]	1 Dose	RCT	12 mo to 17 y (3.9 y)	1	86
Vessey et al (2001) [5]	1 Dose	RCT	12 mo to 12 y (3.6 y)	7	88.5 (95% CI: 80.9– 96.1)
Shinefield et al (2002) [6]	1 Dose	RCT	12 mo to 6 y (1.3 y)	5	Group A: 90.5 (95% CI: 86.2–95.0); group B: 88.9 (83.7–93.7)
Baxter et al (2013) [7]	1 Dose	Prospective cohort	≥12 mo	14	90 (range, 75–90)
Marin et al (2016) [8]	1 Dose	Meta-analysis	Variable	Variable	82 (95% CI: 79–85) (against all varicella)
WHO SAGE (2014) [9]	1 Dose	Literature review	Variable	Variable	Median: 83 (range, 44–100) (against all varicella)

References 3–6 are efficacy and references 7–9 are effectiveness.

Abbreviations: CI, confidence interval; RCT, randomized control trial; SAGE, Strategic Advisory Group of Experts on Immunization; WHO, World Health Organization.

The incremental cost-utility ratios reported in the publication showed marginal differences (at most 15%) between the 2 vaccines across all scenarios and time horizons. Given the sensitivity of incremental cost-utility ratio estimates to small changes in utility gains, results regarding the relative cost-effectiveness of different vaccines need to be interpreted with caution. Sensitivity analyses of relevant data sources for efficacy parameters are warranted to comprehensively test the performance and cost-effectiveness of these vaccines.

## Notes

***Financial support.*** This work was funded by Merck Sharp & Dohme, a subsidiary of Merck & Co.

***Potential conflicts of interest.*** All authors are employees of Merck Sharp & Dohme, a subsidiary of Merck & Co, and M. Pawaskar, M. Pillsbury, M. N. K., and H. L. P. are stockholders of Merck & Co.
